# Development and Validation of an Autophagy Score Signature for the Prediction of Post-operative Survival in Colorectal Cancer

**DOI:** 10.3389/fonc.2019.00878

**Published:** 2019-09-09

**Authors:** Zheng Zhou, Shaobo Mo, Weixing Dai, Zhen Ying, Long Zhang, Wenqiang Xiang, Lingyu Han, Zhimin Wang, Qingguo Li, Renjie Wang, Guoxiang Cai

**Affiliations:** ^1^Department of Colorectal Surgery, Fudan University Shanghai Cancer Center, Shanghai, China; ^2^Department of Oncology, Shanghai Medical College, Fudan University, Shanghai, China; ^3^Department of Cancer Institute, Fudan University Shanghai Cancer Center, Fudan University, Shanghai, China; ^4^Shanghai-MOST Key Laboratory of Health and Disease Genomics, Chinese National Human Genome Center, Shanghai Industrial Technology Institute, Shanghai, China

**Keywords:** colorectal cancer, early relapse, autophagy, signature, nomogram

## Abstract

**Background:** Survival rates for Colorectal cancer (CRC) patients who experienced early relapse have usually been relatively low. Our study aims at developing an autophagy signature that could help to detect early relapse cases in CRC.

**Methods:** Propensity score matching analysis was carried out between patients from the early relapse group and the long-term survival group from GSE39582. For both groups, respectively, global autophagy expression changes were then analyzed to identify the differentially expressed prognostic autophagy related genes by conducting Linear Models for Microarray data method analysis. Then, the multi-gene signature was validated in TCGA and Fudan University Shanghai Cancer Center (FUSCC) cohorts. Time-dependent ROC were used to test the efficiency of this signature feature in predicting the prognosis of CRC patients.

**Results:** 5 autophagy genes were finally identified to build an early relapse classifier. With specific risk score formula, patients were classified into low- or high-risk group. Time-dependent ROC analyses proved its prognostic accuracy, with AUC 0.841 and 0.803 at 1 and 3 years, respectively. Then, we validated its prognostic value in two external validation series (GSE17538 and GSE33113) and proved that the result is indeed significant irrespective of datasets in two external independent validation cohorts (TCGA and FUSCC cohorts). A nomogram was constructed to guide individualized treatment of patients with CRC.

**Conclusions:** The identification of robust autophagy-related features can effectively classify CRC patients into groups with low and high risk of early relapse. This signature may be used to help select high-risk CRC patients who require more aggressive treatment interventions.

## Introduction

As a worldwide malignant tumor, colorectal cancer (CRC) causes 1,632 deaths per day in the United States in 2016, representing ~35% of the CRC patients (1). In 2017, the incidence of CRC in China was about 37.6/100,000, ranking third in all malignant tumors, with about 19.1/100,000 mortality rate at the fourth place (2). The post-operative survival of patients with CRC in different stages varies greatly. For instance, patients with stage IV CRC have a 5-year survival rate of <10% (3, 4), while the 5-year survival rate of stage I patients reaches more than 90% (4). However, there are still no effective methods for the quantitative prognosis of post-operative patients. In the present clinical work, clinicians judge the prognosis of CRC patients with disease stage and pathological features. Vague judging criteria undoubtedly aggravates patients' concerns. Besides, it blocks the efficient operation of clinical work to a certain extent. Therefore, a more precise quantitative prediction tool is urgently needed to assist clinical procedure.

Autophagy is an important mechanism for cells to degrade cytoplasmic components and maintain the stability of the intracellular environment. Including cancer, the occurrence of various diseases is related to the abnormal regulation of autophagy (5, 6). As for malignant tumors, autophagy is closely related to its inflammatory response, drug resistance, and cell death. For example, in terms of tumor occurrence and development, autophagy behavior is closely related to a certain degree. Decreasing expression of p62 and increasing expression of Beclin1 are associated with the development of CRC (7), indicating that autophagic function also plays a role in CRC. On the other hand, autophagy pathway kills cells so that mutations also inhibit the occurrence of tumors. The effect depends on the type of cells, growth-inducing stimulus and mutations. Influence of autophagy on tumors development is determined by cells and tumor micro-environment. However, most studies focus on the choice of treatment options for universal patients, and few studies can achieve the goal of analyzing and processing according to the actual situation of patients.

Nowadays, the most significantly used risk factor for predicting relapse-free survival (RFS) in CRC is based on tumor-node-metastasis (TNM) staging system. For CRC patients at the same tumor stage and with comparable clinical and pathological characteristics, prognosis significantly varies because of the high heterogeneity of CRC. Therefore, the ideal biomarkers or indicators to predict early relapse in CRC patients have always been the research goal for scientists (8, 9). Recently, many malignant tumor related studies have shown that gene signature or multigene expression patterns can predict cancer prognosis favorably (10–12). Especially for CRC patients who have received radical resection surgeries, gene signatures might help to significantly predict prognosis. Nevertheless, there is not enough gene profiling for doctors to find out the RFS associated genes in stage IV CRC.

Researchers were able to conduct a large group of mRNA-specific probes on a commonly used microarray platform (Affymetrix HG-U133 plus 2.0) (13). In this study, we adopted gene expression microarray data from the Gene Expression Omnibus (GEO) and performed mRNA profiling on large cohorts of CRC patients (14). By means of the sample-splitting method and Cox regression analysis, a prognostic five-autophagy related signature was identified from the GSE39582 dataset and validated in the Cancer Genome Atlas (TCGA) database and Fudan University Shanghai Cancer Center (FUSCC) dataset. Based on relapse-free survival, a nomogram was constructed finally as a quantitative prediction tool to evaluate clinical prognosis and to assist clinical procedure.

## Materials and Methods

### The Flowchart of the Study Process

**Figure 1:**
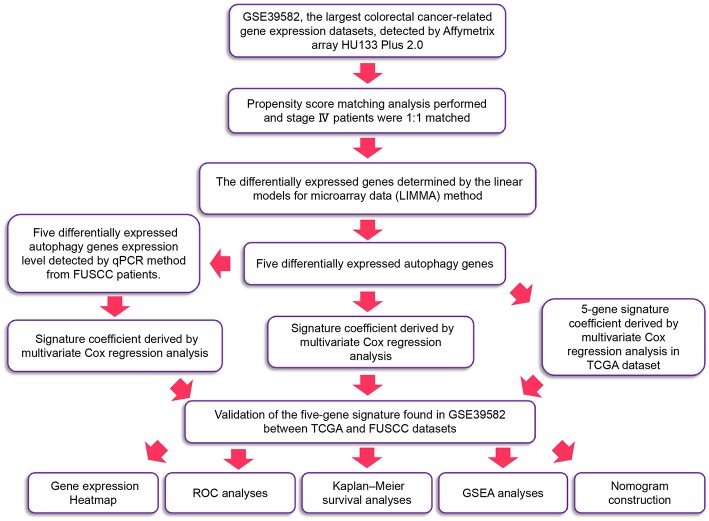
The workflow of identification of CRC survival-related five-autophagy signature.

The detailed workflow is shown in Figure 1. We defined the differentially expressed gene signature from the GSE39582 variables. We then verified it in validation sets through multiple indicators.

### Preprocessing of Transcriptome Data and Autophagy Genes

Raw microarray CRC datasets were obtained from the GEO database (http://www.ncbi.nlm.nih.gov/geo/) and were normalized using Robust Multichip Average (14). All datasets were produced by the Affymetrix HG-U133 plus 2.0 platform. The RNA sequencing datasets of CRC tissues with gene expression levels, including clinicopathological information, were downloaded from TCGA (https://tcga-data.nci.nih.gov/) database. We extracted 232 autophagy genes, reporting in previous research, from the HADb (Human Autophagy Database, http://autophagy.lu/clustering/index.html). The 232 autophagy genes list were shown in Supplementary Table 1.

### Identification of Early Relapse-Associated Genes

Patients suffering from locoregional recurrence or distant metastasis within 1 year after primary resection was classified as early relapsing group. The GSE39582 is the largest colorectal cancer cohort in GEO dataset. We divided CRC cases into the distal and proximal CRC in Table 1 based on GSE39582 dataset. According to the definition, the proximal CRC contains cecum, ascending colon, hepatic flexure, and right transverse colon. The distal CRC contains left transverse colon, splenic flexure, descending colon, sigmoid colon, rectosigmoid junction, and rectum. Stage IV CRC samples receiving radical surgery from GSE39582 were selected and divided into early relapse group and long-term survival group (no relapse after a minimum of 5 years follow-up) (15). Propensity score (PS) matching analysis was performed between the two groups to adjust for T stage and N stage, which were the most significant indexes within TNM staging system. All patients were matched 1:1. Finally, 17 paired patients in GSE39582 set were identified to figure out the changes in the global autophagy genes expression profile between early relapse group and long-term survival group. The analysis of differentially expressed genes (DEGs) between early relapse and long-term survival samples was conducted using the linear models for microarray data (LIMMA) method. The threshold for identification of DEGs was set as *p* < 0.05 and fold change ≥1.2.

**Table 1:** Clinicalopathological features of cases in GSE39582 set before and after propensity score matching.

**Variables**	**Before matching**			**After matching**		
	**Early relapse**	**Long-term survival**	***p*-value**	**Early relapse**	**Long-term survival**	***p*-value**
Age (mean, range)	61.9 (34.0–88.0)	63.1 (34.7–89.4)	0.76	61.9 (34.5–88.0)	63.1 (34.7–86.8)	0.82
Gender			0.03			0.14
Male	19	10		10	7	
Female	12	14		7	10	
T stage			0.08			0.32
T1	0	1		0	0	
T2	0	1		0	0	
T3	18	11		7	9	
T4	13	11		10	8	
N stage			<0.001			0.09
N0	12	1		2	0	
N1	7	15		7	11	
N2	12	8		8	6	
Adjuvant Chemotherapy			0.41			0.48
No	7	8		5	6	
Yes	13	16		3	11	
NA	11	0		9	0	
KRAS status			0.15			0.32
Wild type	16	12		9	7	
Mutation	15	12		8	10	
BRAF status			0.45			0.40
Wild type	26	22		12	15	
Mutation	5	2		5	2	
Location			0.15			0.17
Proximal	7	10		5	10	
Distal	24	14		12	7	
Total	31	24		17	17	

### Statistics for Classification, Prediction, and Validation in GEO Dataset

Using LIMMA analysis, we identified five autophagy related genes differently expressed (*p* < 0.05) in patients with stage IV CRC between early relapse group and long-term survival group. According to the expression values of each selected autophagy genes, a risk score formula was built for each patients, weighted by estimated regression coefficients after multivariate Cox regression analysis (16). With specific risk score formula, patients were divided into high-risk and low-risk groups using the median risk score as the cutoff point. Time-dependent receiver-operating characteristic (ROC) analysis was used to investigate the prognostic or predictive accuracy of each feature and signature. To further confirm the classification reliability of the identified five-autophagy-related-genes signature analyzed by GSE39582, Kaplan–Meier survival curve analyses and log-rank test was performed based on GSE17538 and GSE33113 datasets to evaluate the prognostic significance of the autophagy-related signature.

### TCGA and FUSCC Validation Series

To prove that the result is indeed significant irrespective of datasets enrolled in the study, we validated the result in TCGA and FUSCC validation series. A total of 43 stage IV cases from TCGA set were selected to construct a new risk score formula for each patient, after multivariate Cox regression analysis. ROC analysis was used to investigate the prognostic or predictive accuracy of the signature. Fifty-three primary CRC patients undergoing radical surgery at FUSCC between 2011 and 2012 were retrospectively analyzed in this study. The study design was approved by the Ethical Committee and Institutional Review Board of our cancer center, with written informed consent obtained from all the patients. All the cancer tissues were stored at 80°C. Total RNA extraction and reverse transcription experiments were performed according to the manufacturer's protocol. Real-time PCR was performed on an ABI PRISM 7500 fast Sequence Detection System (Applied Biosystems) using SYBR Green Supermix (Takara). Primers used for amplifying specific genes are presented in Supplementary Table 2. A risk score formula for each patient was constructed. According to the risk score formula and when using the median risk score as the cutoff point, cases were divided into low-risk and high-risk groups. Survival differences between the two groups were assessed via the Kaplan–Meier and compared using the log-rank test.

### Construction of the Nomogram

In the GSE39582 cohort, variables achieving *p* < 0.05 were selected for multivariable analyses via the Cox regression model. Eventually, to assist clinical procedure, a nomogram, integrated the five-autophagy-related signature and clinicopathological risk factors, was constructed as a quantitative prediction tool to evaluate clinical prognosis.

### Functional Enrichment Analysis

Functional enrichment analyses for autophagy-related genes in the signature set were performed through the Database for Annotation, Visualization and Integrated Discovery (DAVID, https://david.ncifcrf.gov/) (17). Gene Set Enrichment Analysis (GSEA) was performed by the JAVA program (http://software.broadinstitute.org/gsea/downloads.jsp) using the MSigDB C2 Canonical pathways gene set collection, containing 1,320 gene sets. Gene sets with a false discovery rate (FDR) value <0.05 after performing 1,000 permutations were considered to be significantly enriched (18).

### Statistical Analysis

All statistical analyses were performed with the use of R (version 3.5.1, www.r-project.org). All statistical tests were two-sided, and *p* < 0.05 were considered statistically significant.

## Results

### Preparation of CRC Datasets

A total of 1,485 patients were identified and fully studied, which included 497 patients from GSE39582, 157 cases from GSE17538, 89 cases from GSE33113, 689 cases from TCGA database, and 53 patients from FUSCC set. Cases missing necessary clinicopathological or follow-up data were excluded.

### Development of Early Relapse Signature From GSE39582 Set

Stage IV CRC patients from GSE39582 set were divided into early relapse group and long-term survival group. Before and after PS matching, clinicopathological features of this patients are described in Table 1. After PS matching, there were no significant differences in age, sex, T stage, and N stage between early relapse and long-term survival groups (Table 1). The analysis of discrepantly expressed autophagy genes (DEAGs) between the two groups was performed using LIMMA method. *P* < 0.05 and fold change ≥1.2 were perceived as statistically significant as to the identification of DEAGs. Then, five autophagy-related genes were found differentially expressed between two groups. Using Cox proportional hazards regression modeling, we derived a 5-autophagy-related genes signature to calculate the risk score for each patient based on the expression levels of the 5 genes weighted by their regression coefficients: Risk score = (0.81995105 * expression level of CAPN10) + (−0.03919814 * expression level of DAPK2) + (0.82646924 * expression level of DNAJB9) + (1.06760178 * expression level of GNAI3) + (0.21783501 * expression level of PPP1R15A).

### The Prognostic Value of 5-Autophagy-Related Signature in GSE39582 and Two External Validation Series

**Figure 2:**
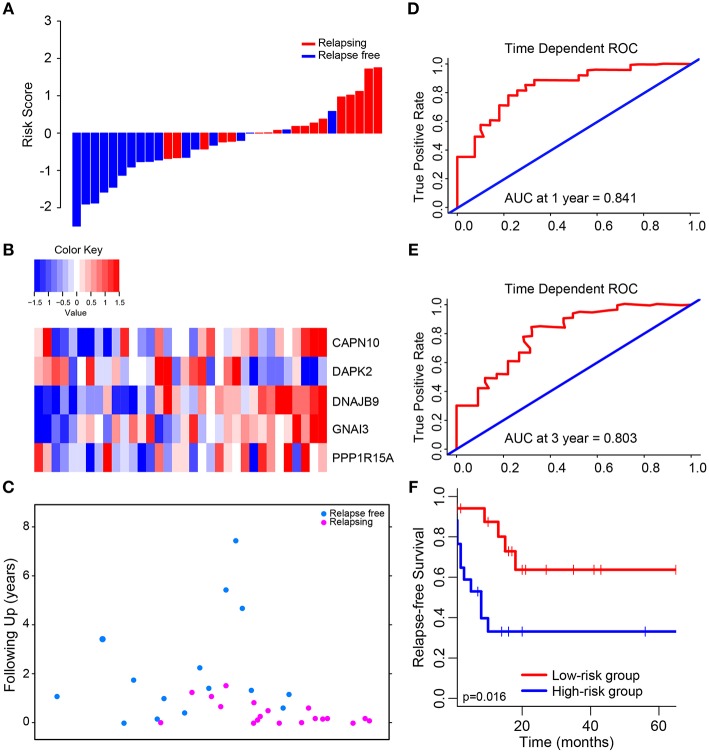
Determination and analysis of the 5-autophagy-related signature in GSE39582 cohort. **(A)** The distribution of patients' risk score and recurrence status. **(B)** The expression pattern of the 5-autophagy-related signature. **(C)** The survival status of CRC patients. **(D)** Time dependent ROC curves at 1 year. **(E)** Time dependent ROC curves at 3 years. **(F)** Kaplan–Meier survival curves of RFS between high-risk and low-risk patients in GSE39582 cohort.

The median risk score was used in patients from GSE39582 set to divide them into low-risk group (*N* = 17) or high-risk group (*N* = 17) as a cut-off point. The distribution of risk scores and survival status is shown in Figure 2, which suggested that patients with lower risk scores generally had better survival than those with higher risk scores. Time-dependent ROC analyses at 1 and 3 years were conducted to assess the prognostic accuracy of the 5-autophagy-based classifier. The AUC was 0.841 and 0.803 at the survival time of 1 and 3 years, respectively, in GSE39582 (Figures 2D,E). The RFS rates for patients in low-risk group were 80.1% at 1 year and 63.7% at 3 year, compared with 39.7%, and 33.1% in patients in high risk group, respectively (*p* = 0.016, Figure 2F).

Then, we compared the expression level of each autophagy related gene generated from GSE39582 between high risk group and low risk group. There were significant differences in the expression level of these five genes between high and low risk groups (Supplementary Figure 1), in which the expression level of CAPN10, DNAJB9, GNAI3, and PPP1R15A in high-risk group was higher than that in low-risk group, while DAPK2 was the opposite.

**Figure 3:**
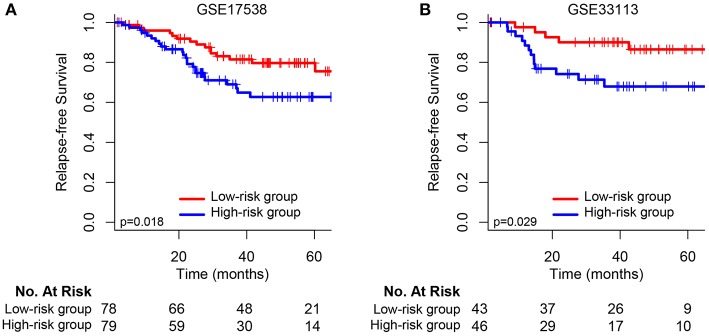
Kaplan–Meier survival analysis between patients at low and high risk of relapse in two independent external validation sets: **(A)** GSE17538 and **(B)** GSE33113.

We validated and confirmed the predictive power of the signature in another two independent datasets (GSE17538 and GSE33113). Using the established risk score formula, each of the cases was divided into high-risk and low-risk group. Consistent with the above findings, significantly different outcomes were found between the high-risk group and the low-risk group. The HR for relapse-free survival of high-group vs. low-group was 2.118 (95% CI: 1.119–4.007, *p* = 0.018) and 3.002 (95% CI: 1.067–8.444, *p* = 0.029) for GSE17538 (Figure 3A) and GSE33113 (Figure 3B), respectively. Subgroup analyses based on TP53 mutation status, KRAS mutation status and adjuvant chemotherapy suggested that high-risk patients in each subgroup were inclined to have significantly unfavorable RFS (Supplementary Figure 2).

### External Validation of the Signature in TCGA and FUSCC Datasets

We have developed and validated a 5-autophagy-related signature based on the cases from GEO dataset (GSE39582, GSE17538, and GSE33113). In order to prove that the result is of significance, regardless of datasets participating in the study, we then validated the result in TCGA and FUSCC cohorts. Baseline characteristics of CRC patients in TCGA and FUSCC validation cohorts were shown in Table 2.

**Table 2:** Baseline characteristics of CRC patients in TCGA and FUSCC validation cohorts.

**Variables**	**TCGA cohort**	**FUSCC cohort**
Age (mean, range)	62.4 (35–90)	60.4 (45–78)
**Gender**		
Male	25	25
Female	18	28
**T stage**		
T1	1	2
T2	1	3
T3	29	30
T4	12	18
**N stage**		
N0	1	23
N1	25	18
N2	17	12
**Adjuvant therapy**		
No	7	15
Yes	8	36
NA	28	2
**KRAS status**		
Wild type	7	20
Mutation	4	16
NA	32	17
**Tumor location**		
Colon	34	24
Rectum	9	29
Total	43	53

*CRC, colorectal cancer; TCGA, The Cancer Genome Atlas; FUSCC, Fudan University Shanghai Cancer Center*.

**Figure 4:**
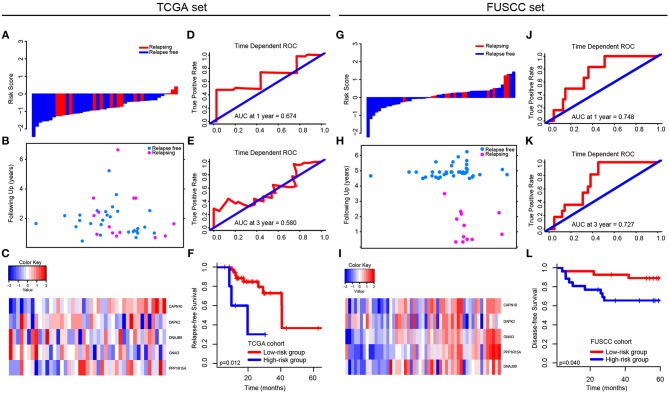
Validation of the 5-autophagy-related signature in TCGA and FUSCC independent cohorts: The distributions of the risk score **(A,G)**, survival status **(B,H)**, and 5 autophagy expression values associated with CRC patients **(C,I)**. Time dependent ROC curves at 1 year **(D,J)** and 3 years **(E,K)**. Kaplan–Meier survival curves of RFS between high-risk and low-risk patients in TCGA cohort **(F)**. Kaplan–Meier survival curves of DFS between high-risk and low-risk patients in FUSCC cohort **(L)**.

In TCGA set, we analyzed the different expression level of the five genes between tumor and non-tumor tissue in TCGA, which was shown in Supplementary Figure 3. The correlation between the expression level of each gene and CRC patients' outcomes was then analyzed (Supplementary Figure 4). Only the expression level of CAPN10 and PPP1R15A were related to CRC patients' prognosis (*p* = 0.042 and *p* = 0.034, respectively), indicating that a single gene cannot predict the prognosis of CRC patients. Therefore, using Cox proportional hazards regression modeling, we calculated the risk score for each patient based on the expression levels of the 5-autophagy-related genes weighted by their regression coefficients: Risk score = (1.30745099 * status of CAPN10) + (−0.12398368 * status of DAPK2) + (0.41541350 * status of DNAJB9) + (0.04859172 * status of GNAI3) + (0.43307198 * status of PPP1R15A) (Figures 4A–C). Similar to the results generated by GSE39582, time-dependent ROC analyses at 1 and 3 years were conducted to assess the prognostic accuracy of the 5-autophagy-based classifier, with AUC 0.674 and 0.580 at the survival time of 1 and 3 years, respectively, in TCGA set (Figures 4D,E). The RFS rates for patients in low-risk group were 88.5% at 1 year and 60.7% at 3 years, compared with 60.0%, and 30.0% in patients in high risk group, respectively (*p* = 0.012, Figure 4F).

In FUSCC cohort, based on individual expression status of the 5 autophagy genes analyzed by Real-time PCR, we used Cox proportional hazards regression modeling to derive a formula, in which we can calculate the risk score for each patient to access their risk of disease recurrence: Risk score = (0.006692504 * status of CAPN10) + (−0.219417213 * status of DAPK2) + (0.016421389 * status of DNAJB9) + (0.320954863 * status of GNAI3) + (0.238786909 * status of PPP1R15A). Distribution of the 5 autophagy genes expression levels is based on risk scores, and the expression pattern of 5 autophagy biomarkers among the 53 CRC patients belonging to the FUSCC cohort are shown in Figures 4G–I. The AUC was 0.748 and 0.727 at 1 and 3 years, respectively (Figures 4J,K). Further, the survival time of the high-risk group was significantly shorter than that of the low-risk group (*p* = 0.040, Figure 4L).

### Identification of the 5-Autophagy-Related Signature Associated Biological Pathways

**Figure 5:**
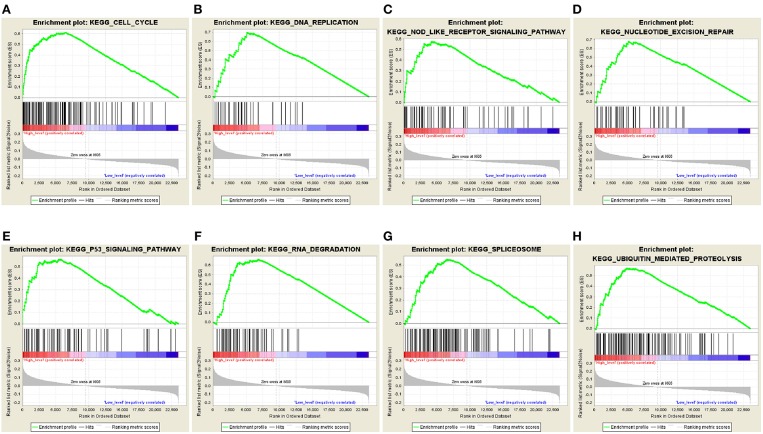
The GSEA analysis results in GSE39582. GSEA validated enhanced activity of **(A)** “Cell cycle,” **(B)** “DNA replication,” **(C)** “Nod like receptor signaling pathway,” **(D)** “Nucleotide excision repair,” **(E)** “P53 signaling pathway,” **(F)** “RNA degradation,” **(G)** “Spliceosome,” and **(H)** “Ubiquitin mediated proteolysis” in high risk score group.

The predictive power of the 5-autophagy-related signature in predicting recurrence risk of CRC patients could be attributed to their crucial roles in tumor development or metastases. Therefore, we performed GSEA analyses to identify autophagy associated pathways in GEO gene expression datasets. Classified by the 5-autophagy-related signature, genes were ranked according to differential significance between the high- and low-risk groups. Only when the nominal *p* < 0.005 and the FDR < 0.05 were achieved, based on a canonical pathways gene set from the MSigDB database, could gene sets be considered significantly enriched. In the GSEA enrichment results, we observed that the “Cell cycle,” “DNA replication,” “Nod like receptor signaling pathway,” “Nucleotide excision repair,” “P53 signaling pathway,” “RNA degradation,” “Spliceosome,” and “Ubiquitin mediated proteolysis” pathways were enriched in the high risk groups (Figures 5A–H). Several studies have indicated that these pathways were associated with the development of CRC. Briefly, the GSEA analyses results implied that the 5-autophagy-related signature was associated with the CRC development and progress.

### Setting Up a Nomogram as Prognostic Prediction Model

**Figure 6:**
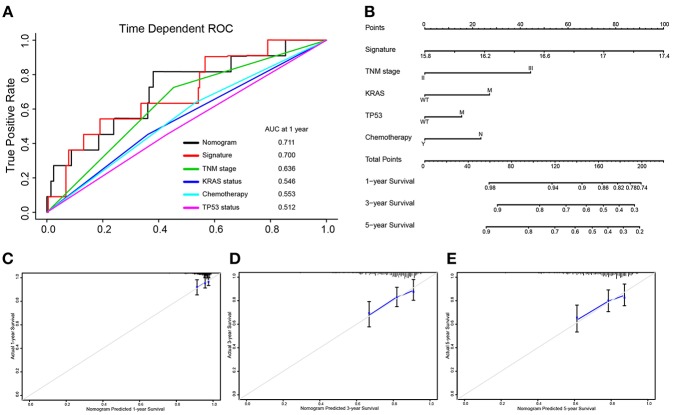
Nomograms convey the results of prognostic models using the 5-autophagy-related signature and four clinicopathological characteristics to predict RFS of patients with colon cancer. The AUC at 1-year prediction was 0.711 **(A,B)**. The x-axis is nomogram-predicted probability of survival and y-axis is actual survival. The reference line is 45° and indicates perfect calibration [**C** (1-year), **D** (3-year), **E** (5-year)].

Integrated the five-autophagy-related classifier and four clinical pathology risk factors, we constructed a nomogram to meet the needs of clinician to quantify the possible risk of cancer relapse. Nomogram can be interpreted by a summary of the points assigned to each variable, displayed at the top of the scale. The total score can be converted to the lowest-scale patient with an RFS probability of 1 year, 3 years, and 5 years (Figure 6B). To confirm that the nomogram prediction model had higher efficacy in predicting early relapse, time-dependent ROC was used, which suggested that the nomogram had the highest prognostic accuracy by a significant amount. The AUC at 1-year prediction was 0.711 (Figure 6A). Calibration curves of the nomogram revealed no deviations from the reference line and no recalibration required [Figure 6C (1-year), Figure 6D (3-year), Figure 6E (5-year)].

## Discussion

Postoperative relapse is closely related to patients' survival. Currently, prediction results for stage IV CRC usually do not correspond with their actual prognosis situations because their respective genetic and epigenetic conditions vary from person to person (19). Proper biomarkers to reveal early postoperative disease progression would enhance the effectiveness of standard TNM classification and supporting physicians in developing more effective treatment strategies in the early stages of CRC patients' treatment.

Previous studies have tried to identify postoperative molecular markers for detection of postoperative RFS in CRC, especially in stage I–III CRC (20, 21). However, research has neglected stage IV metastatic CRC, which is of great importance in both quantity and prognosis of CRC. Besides, biomarker related research is the lack of attention within stage IV CRC patients. Previous studies have demonstrated that autophagy is closely related to malignant tumors' inflammatory response, drug resistance, and cell death (22). Hence, we focused on the prognosis of stage IV CRC and autophagic expression profiles to find autophagy related biomarkers that could be used to predict the prognosis of CRC.

In the current study, a novel prognostic classifier based on five autophagy related genes was developed to improve the prediction of early relapse and prediction of RFS for stage IV CRC patients after surgical resection. By applying the five-autophagy-related signature to CRC patients, a clear separation was observed in the survival curves between low- and high-risk patients. Afterwards, it was successfully validated in the external cohorts of TCGA and FUSCC databases, indicating the good reproducibility of this signature. To confirm whether this conclusion could be broadened to II–IV CRC patients, validation analyses were continued in stage II–III CRC patients. We performed Kaplan–Meier survival curve analyses and log-rank test based on GSE17538 and GSE33113 datasets to evaluate the prognostic significance of the 5-autophagy-related signature. Furthermore, the time-dependent ROC suggested that this five-mRNA signature has considerable prognostic accuracy in predicting tumor relapse within the first year and third year after initial resection of CRC. Using this five-autophagy-related-gene signature, TNM stage, Adjuvant Chemotherapy, KRAS, and TP53 mutation, a nomogram was constructed to predict the 1-, 3- and 5-year RFS probabilities after curative surgery. The results were inspiring, which proved that this signature is extensively applicable in patients with stage II-IV CRC. Compared to previous research, this study has the following strengths. Firstly, stage I patients were excluded to decrease the bias when completing autophagy related gene profiling. Secondly, the results were identified from the stage IV CRC patients and then validated in stage II–IV CRC patients. Thirdly, our study group identified and validated, for the first time, a autophagy-related signature from three different datasets: GEO, TCGA, and FUSCC datasets. Lastly, we also identified the 5-autophagy-related signature associated biological pathways, which were proven to be associated with the CRC development and progress. Hence, our study identified a five-autophagy-related-gene signature that could help identify patients with a high risk of early relapse and guide individualized treatment of patients with CRC, which is credible to be applied to clinical application.

All genes in the signature are strongly correlated to cancer, as has been empirically demonstrated by previous experiments. Of these, CAPN10, PPP1R15A, and DAPK2 have been previously reported to have a clear correlation with CRC. Some scientists identified that the calpain-10 (CAPN10) gene is the first candidate type 2 diabetes mellitus (T2DM) gene, which is associated with decreased glucose tolerance and insulin resistance (IR) phenotypes by genome-wide linkage and positional cloning. Previous studies suggested that CAPN10 is important for IR, which is relevant to CRC risk (23). Consequently, results from foregoing study also support our hypothesis that, for patients whose expression level of CAPN10 is low and clinicopathological characteristics are comparable, survival chances are higher. Protein phosphatase 1 regulatory subunit 15A (PPP1R15A) showed the highest correlation with FRB-driven tumor IR and drug responses. Some PPP1R15A mutations may improve the prediction of metastasis CRC patients sensitive to bevacizumab regimens (24). Death-associated protein kinase 2 (DAPK2) regulates cytoskeleton-associated proteins to suppress cancer cells directed migration. We presumed that DAPK expression decreased toward the tumor invasion front, which resulted in patients better survival (25). In addition to CRC, tumor cell migration and invasion is inhibited by the Guanine nucleotide binding protein, alpha inhibiting activity polypeptide 3 (GNAI3), in hepatocellular carcinoma (26). Another piece of research showed that DNAJB9 inhibited the pro-apoptotic function and belongs to the group of negative feedback regulators of p53, which might cause uncontrolled cell growth (27).

Moreover, GSEA analyses analyzed the differences between high- and low-risk groups, stratified by the five-mRNA signature. We have identified several CRC-related pathways, such as “Cell cycle,” “DNA replication,” “Nod like receptor signaling pathway,” “Nucleotide excision repair,” “P53 signaling pathway,” “RNA degradation,” “Spliceosome,” and “Ubiquitin mediated proteolysis” pathways, which were significantly enriched in the high risk group. It is also suggested that the 5-autophagy-related signature may be concerned with CRC-related biological pathways and their functional dysregulations may lead to CRC relapse in GSEA enrichment results.

There are indeed some limitations in this study. On the one hand, our research was based on the public data which lacked several important clinicopathological features, such as specific metastatic tumor location, and has not been prospectively tested in clinical trials. On the other hand, the underlying mechanism of how the identified 5-autophagy-related genes function in the early relapse of CRC still requires further research to be unveiled.

## Conclusion

In conclusion, we developed a five-autophagy-related mRNA signature composed of various regulation mRNA that effectively classify CRC patients into low-risk and high- risk groups of postoperative relapses. Application of the signature in clinical treatments should also be further observed to verify the validity of our findings.

## Data Availability

All data that support this manuscript are made available by the authors, without undue reservation, to any qualified researcher.

## Ethics Statement

This study design was approved by the Ethical Committee and Institutional Review Board of Fudan University Shanghai Cancer Center, with written informed consent obtained from all the patients.

## Author Contributions

ZZ, SM, and WD had the idea for this study. QL and RW supervised the acquisition of the data. LZ and ZY undertook the statistical analysis. WX, LH, and ZW provided statistical advice. All authors contributed to interpretation of the results. ZZ, SM, and GC wrote the article and other authors contributed to the content. All authors approved the final version of the manuscript, including the authorship list.

### Conflict of Interest Statement

The authors declare that the research was conducted in the absence of any commercial or financial relationships that could be construed as a potential conflict of interest.

## Supplementary Material

Supplementary Table 1 The 232 autophagy genes list.

Supplementary Table 2 Primers used for PCR validation.

Supplementary Figure 1 The comparison of the expression level of each autophagy related genes generated from GSE39582 between high risk group and low risk group. **p* < 0.05; ***p* < 0.01; ****p* < 0.001.

Supplementary Figure 2 Subgroup analyses based on TP53 mutation status **(A,B)**, KRAS mutation status **(C,D)**, and adjuvant chemotherapy **(E,F)**.

Supplementary Figure 3 The different expression level of each autophagy related genes between tumor and non-tumor tissue in TCGA. ***p* < 0.01; ****p* < 0.001; ns, not significant.

Supplementary Figure 4 The correlation between the expression level of CAPN10 **(A)**, DAPK2 **(B)**, DNAJB9 **(C)**, GNAI3 **(D)**, PPP1R15A **(E)** and CRC patients' outcomes.
