# Supplementary material for: Development and Validation of an Autophagy Score Signature for the Prediction of Post-operative Survival in Colorectal Cancer
Source: Front Oncol. 2019 Sep 9;9:878. doi: 10.3389/fonc.2019.00878 (PMC6746211; doi:10.3389/fonc.2019.00878)
Supplement: Supplementary Table 1 — The 232 autophagy genes list. [file Table_1.docx]

| **Supplementary Table 1. Primers used for PCR validation** | |
| --- | --- |
| Gene | Forward and Reverse primer |
| CAPN10 | F: 5' CTTCTGCGACTTGTCTACGCC 3' |
|  | R: 5' GTGTGGCACAAATCTCCTGG 3' |
| DAPK2 | F: 5' ATTGCTCACTTTGATCTCAAGCC 3' |
|  | R: 5' AAATTCCGGCGTCCCAAAAAT 3' |
| DNAJB9 | F: 5' TCTTAGGTGTGCCAAAATCGG 3' |
|  | R: 5' TGTCAGGGTGGTACTTCATGG 3' |
| GNAI3 | F: 5' GACGGCTAAAGATTGACTTTGGG 3' |
|  | R: 5' CCGTTTAATCACTCCTGCTAGTT 3' |
| PPP1R15A | F: 5' ATGATGGCATGTATGGTGAGC 3' |
|  | R: 5' AACCTTGCAGTGTCCTTATCAG 3' |
| β-actin | F: 5' CATGTACGTTGCTATCCAGGC 3' |
|  | R: 5' CTCCTTAATGTCACGCACGAT 3' |
