# Supplementary figures and images for: Development and Validation of an Autophagy Score Signature for the Prediction of Post-operative Survival in Colorectal Cancer
Source: Front Oncol. 2019 Sep 9;9:878. doi: 10.3389/fonc.2019.00878 (PMC6746211; doi:10.3389/fonc.2019.00878)

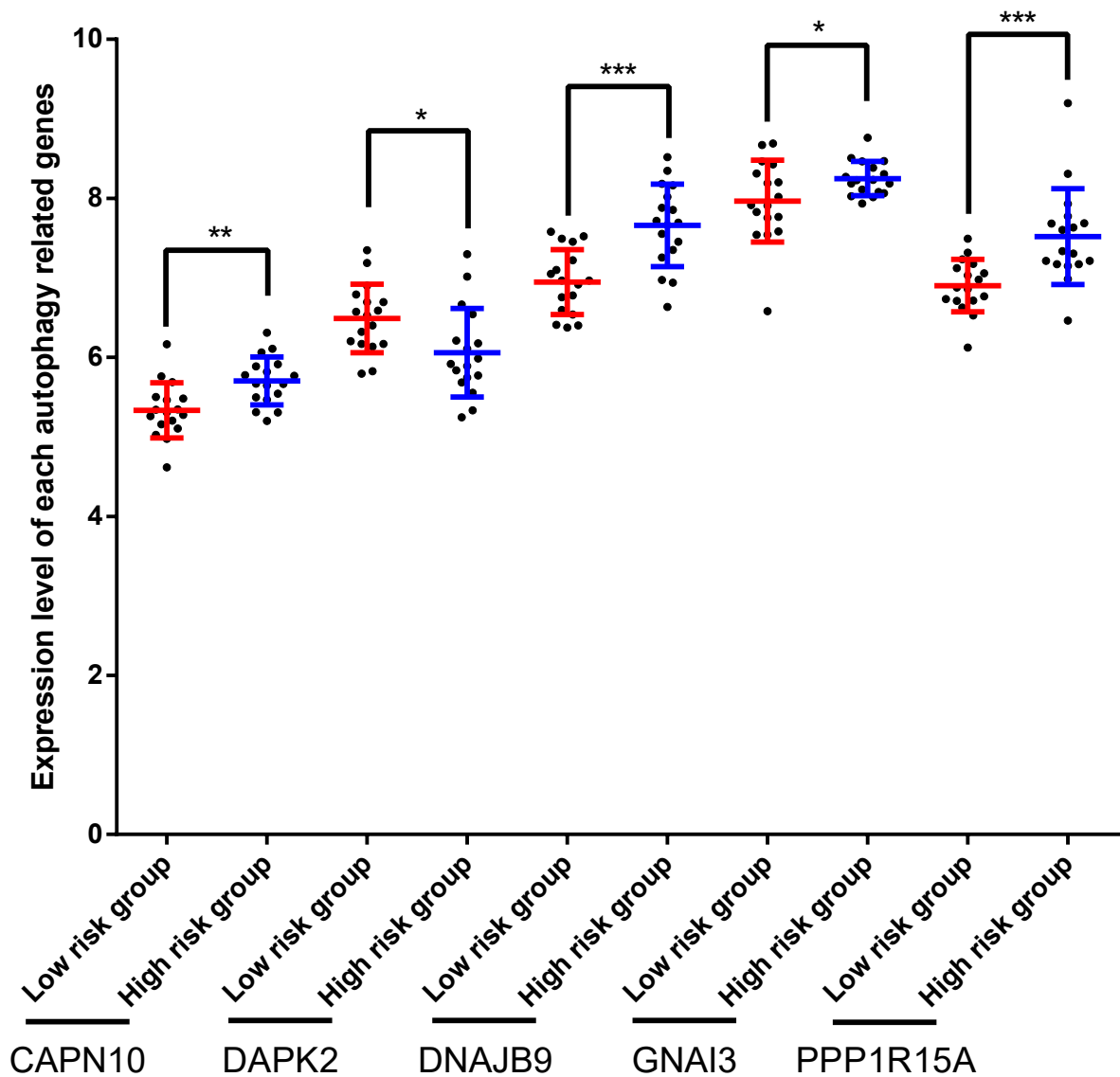

Supplement: Supplementary Figure 1 — The comparison of the expression level of each autophagy related genes generated from GSE39582 between high risk group and low risk group. *p < 0.05; **p < 0.01; ***p < 0.001. [file Image_1.pdf]

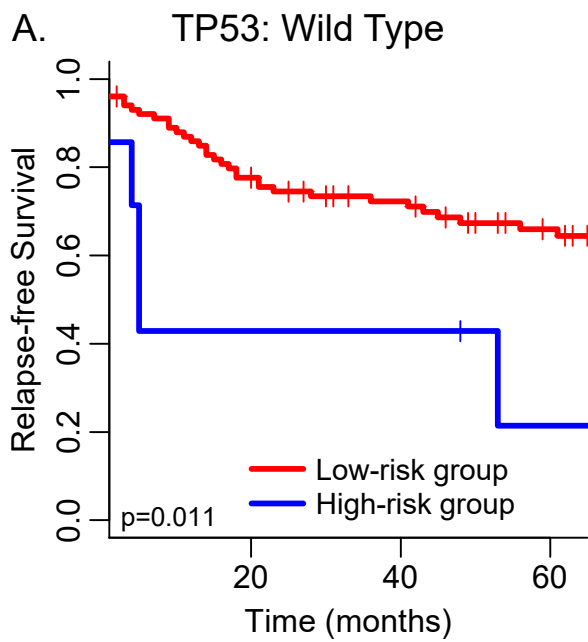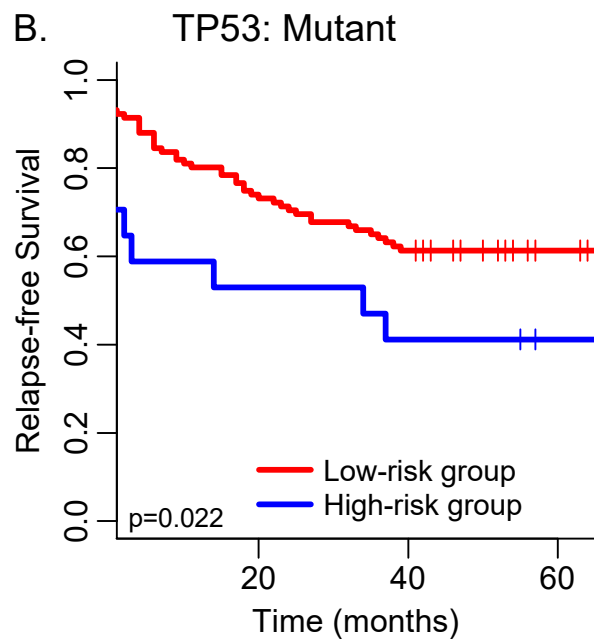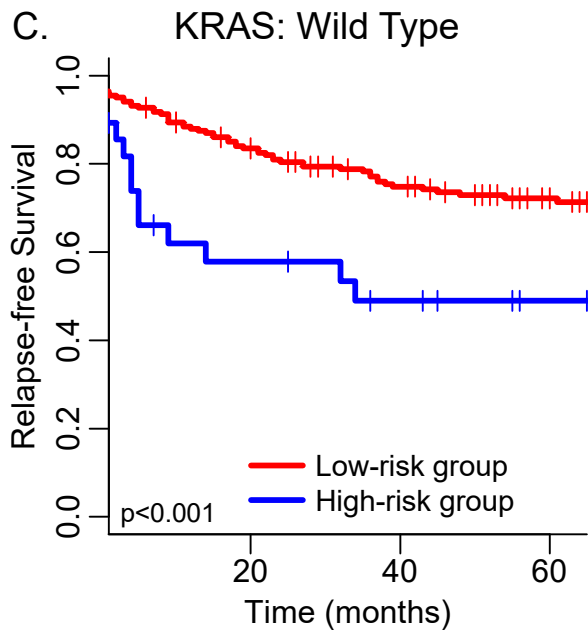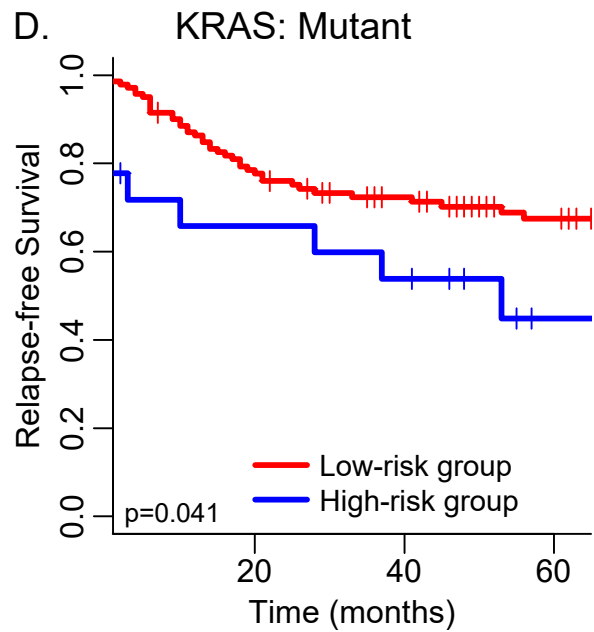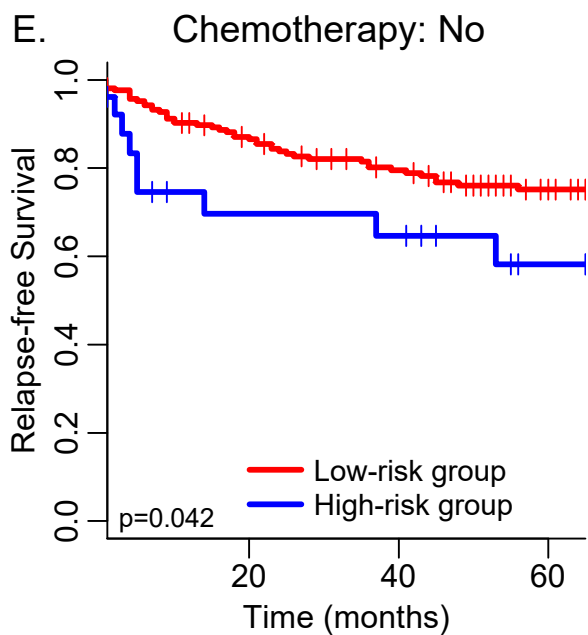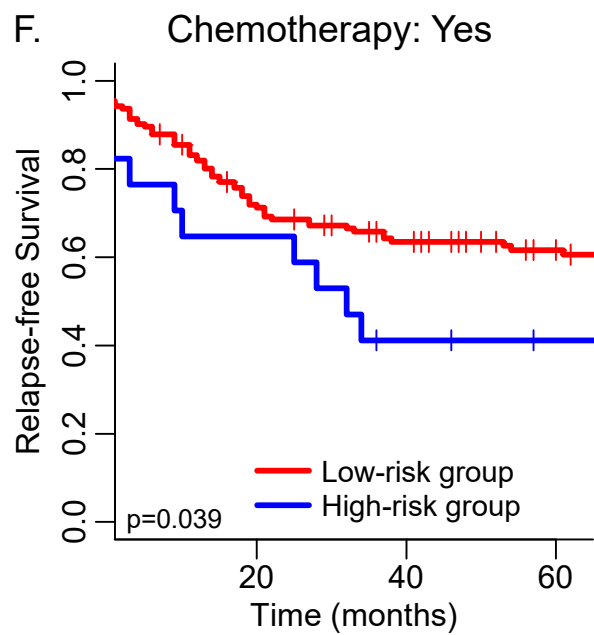

Supplement: Supplementary Figure 2 — Subgroup analyses based on TP53 mutation status (A,B), KRAS mutation status (C,D), and adjuvant chemotherapy (E,F). [file Image_2.pdf]

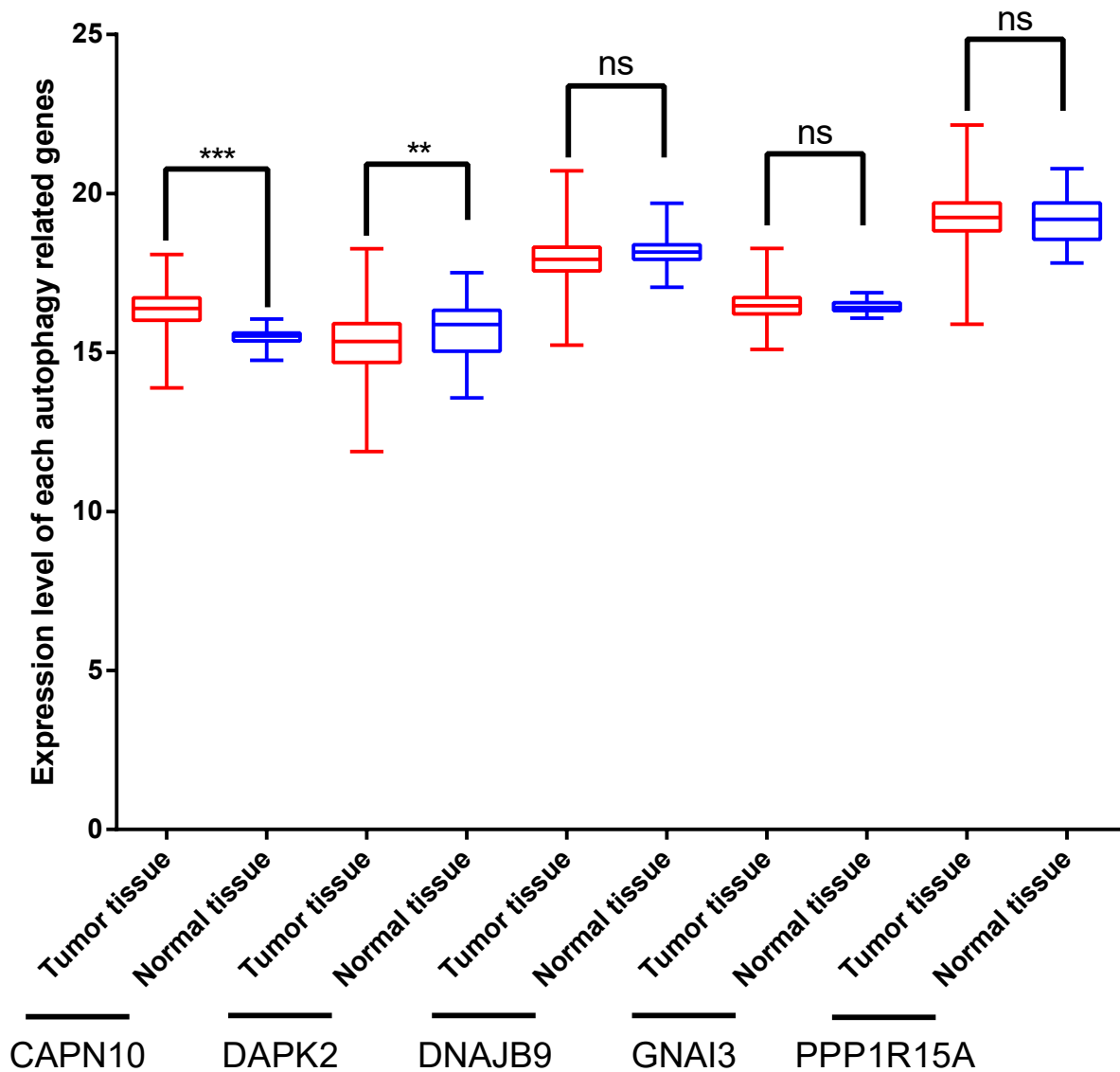

Supplement: Supplementary Figure 3 — The different expression level of each autophagy related genes between tumor and non-tumor tissue in TCGA. **p < 0.01; ***p < 0.001; ns, not significant. [file Image_3.pdf]

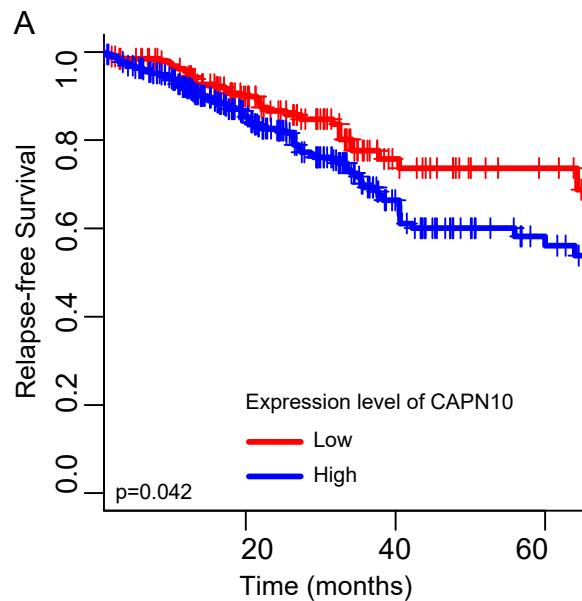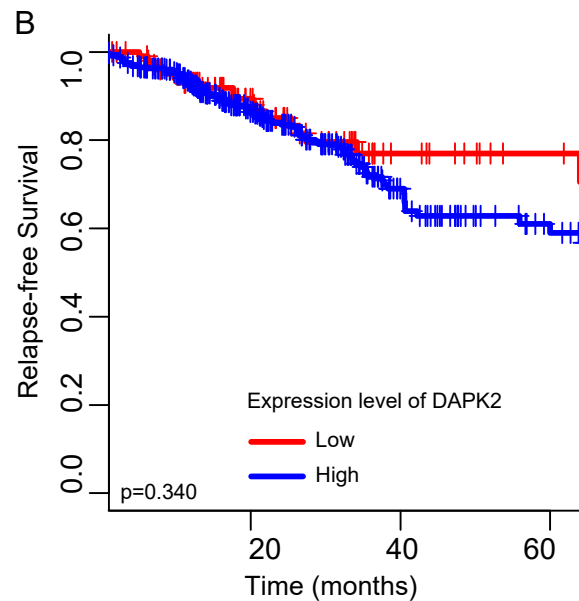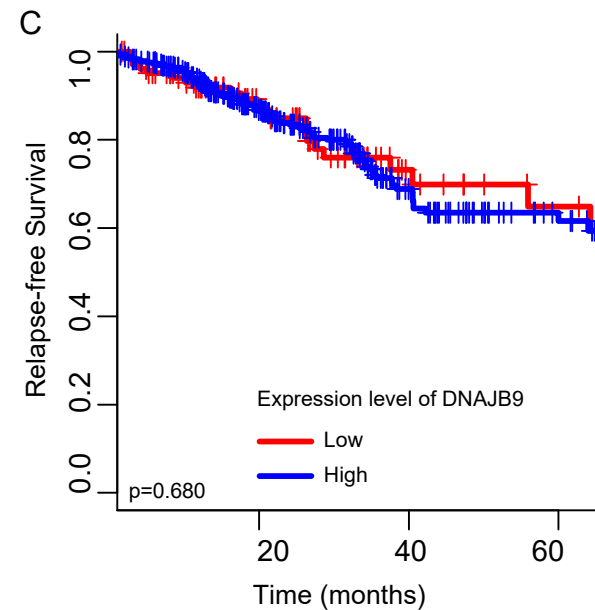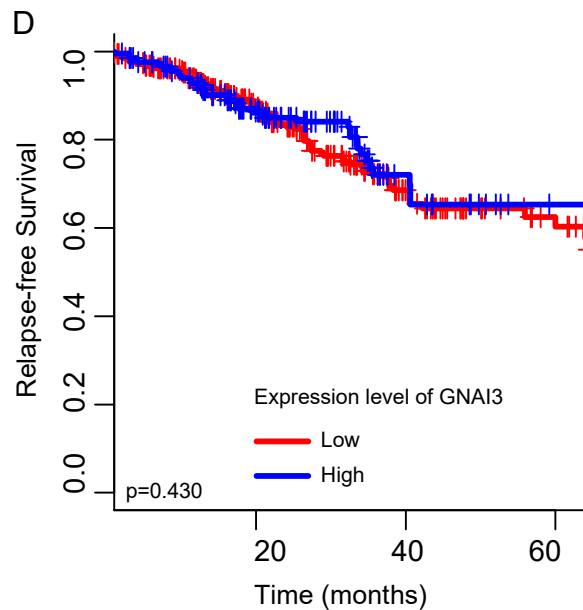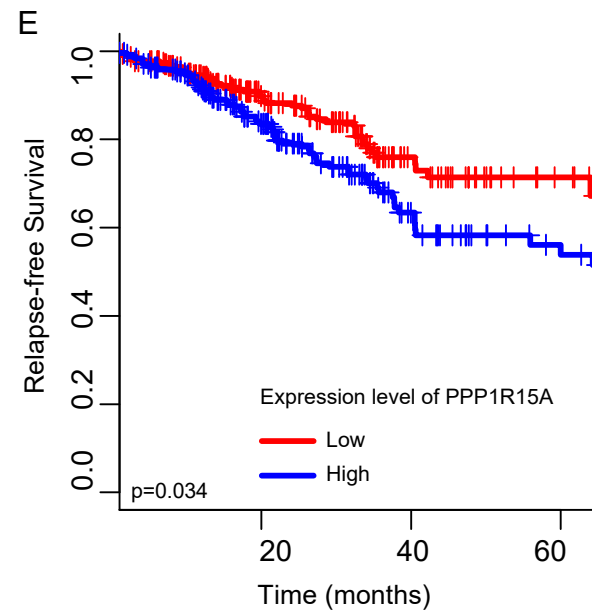

Supplement: Supplementary Figure 4 — The correlation between the expression level of CAPN10 (A), DAPK2 (B), DNAJB9 (C), GNAI3 (D), PPP1R15A (E) and CRC patients' outcomes. [file Image_4.pdf]
